# The *OXTR* Single-Nucleotide Polymorphism rs53576 Moderates the Impact of Childhood Maltreatment on Empathy for Social Pain in Female Participants: Evidence for Differential Susceptibility

**DOI:** 10.3389/fpsyt.2018.00359

**Published:** 2018-08-08

**Authors:** Vera Flasbeck, Dirk Moser, Robert Kumsta, Martin Brüne

**Affiliations:** ^1^Division of Cognitive Neuropsychiatry and Psychiatric Preventive Medicine, Department of Psychiatry, Psychotherapy and Preventive Medicine, LWL University Hospital, Ruhr University Bochum, Bochum, Germany; ^2^Department of Genetic Psychology, Faculty of Psychology, Ruhr University Bochum, Bochum, Germany

**Keywords:** empathy, oxytocin, rs53576, social pain, childhood trauma, differential susceptibility, borderline personality disorder

## Abstract

Previous research has associated genetic variations of the oxytocin receptor with individual differences in human social behavior. Specifically, homozygous carriers of the G-allele of the single nucleotide polymorphism rs53576 have been reported to display more trust, empathy, and prosocial behavior and were less sensitive toward stress and maltreatment during childhood when compared to A-allele carriers. With regard to Borderline Personality Disorder (BPD), a psychiatric condition that is often associated with the experience of childhood adversity, it has been suggested that A-allele carriers are more vulnerable to developing psychopathological signs and symptoms. In the present study we investigated whether childhood trauma, as assessed by the Childhood Trauma Questionnaire (CTQ), affects empathy for somatic and psychological pain, and how this is moderated by genotype, in a sample of 302 individuals (148 of whom were diagnosed with BPD). We found a three-way interaction between genotype, group and pain condition. *Posthoc* comparisons revealed that patients with BPD carrying at least one A-allele, rated psychological pain as more intense compared to controls, whereas no difference between groups emerged in GG homozygotes. Moreover, a moderating effect of genotype appeared on the impact of childhood trauma on empathy for psychological pain. In addition, a positive correlation of CTQ scores and empathy appeared only in A-allele carriers (GA + AA), independent of diagnosis. Together, A-allele carriers, especially those with BPD, seemed to be responsive to the impact of adversity on empathy-for-pain, while GG homozygotes were not, which is compatible with the idea of differential susceptibility.

## Introduction

The ability to empathize with others is a key feature of human social interaction and involves the ability to share another's emotion and to reflect upon the underlying causes of that emotion (1–3). There is substantial inter-individual variability in empathy, which is explained by a number of factors, including early experiences with important attachment figures as well as genetic variation (3). Pervasive deficits in empathy occur, for instance, in psychopathy, autism, schizophrenia, fronto-temporal dementia, and antisocial personality disorders (4–9). An increasing body of research suggests that variation of genes coding for key players involved in oxytocin signaling substantially contribute to individual differences in empathy (10). Oxytocin is a neuropeptide that is well-conserved in the mammalian lineage, and is suggested to play a key role in social and reproductive behavior (11, 12). Research into polymorphic variations of the oxytocin receptor gene (*OXTR*) has demonstrated that several variations of the receptor gene are associated with empathic concern for others. The most extensively researched single-nucleotide polymorphism (SNP) of the *OXTR* is the rs53576, a SNP in the third of four introns (13). Uzefovsky and colleagues reported that the presence of one rs53576 A-allele predicted lower emotional empathy in psychologically healthy humans (14). Conversely, rs53576 GG carriers showed more pronounced sympathetic nervous system reactivity and arousal to another's distress, and reported higher empathic concern compared with A-allele carriers (15, 16). Moreover, rs53576 GG homozygotes were found to show enhanced empathy in the “Reading the Mind in the Eyes” Test, which examined the ability to infer another's mental from the eye region (17). Other studies further demonstrated ethnicity-specific effects, whereby G-allele carriers in an American sample, but not Korean participants, were seeking more intensely emotional social support than AA homozygotes when exposed to social stress (18). Along similar lines, a study in German participants reported that social support reduced the stress response only in G-allele carriers (19). Two meta-analyses support the notion of increased empathy and higher general sociality in G-allele carriers. Gong and colleagues showed that Chinese individuals with one or two copies of the G-allele showed enhanced fantasizing about another's feelings. The additional meta-analysis confirmed these findings in Europeans and Asians (20). Likewise, the meta-analysis by Li et al. (21) investigated two indices of sociality, namely general sociality, which means how individuals respond to others in general, and sociality in close relationships. They reported an association of rs53576 genotype with general sociality, but not close relationships. GG homozygotes showed higher sociality than A-allele carriers, which was not affected by ethnicity. In sum, the GG genotypes are assumed to show enhanced empathy and responsiveness to social support, as well as attenuated stress sensitivity compared to A-allele carriers [for a review, see (22)].

With regard to psychopathological conditions, variation of the rs53576 genotype is assumed to be associated with autism (23, 24) and schizophrenia (25). Moreover, a previous study found an association of rs53576 genotype with risk for borderline symptomatology when associated with childhood maltreatment (26). More precisely, BPD symptom severity was more pronounced in A-allele carriers who grew up in families with depressed mothers, whereas the risk for expressing borderline symptoms was reduced in more supporting family conditions. GG carriers, by comparison, were unresponsive to environmental quality (27). Similar, McInnis and colleagues reported a moderating role of the genotype in the association of unsupportive parenting and coping strategies which affects the development of depressive symptoms. That is, unsupportive interactions were related to increased emotion-focused coping only in A-allele carriers, which was related to more severe depressive symptoms, pointing toward a role of the A-allele in stress-related coping deficits (28). The same group also reported that rejection sensitivity, which was related to depressive scores, was associated with multiple group membership in individuals carrying the A-allele (29). In contrast, they also found that GG carriers were more sensitive to social exclusion and showed higher stress responses compared to A-allele carriers (30). These results lend support to speculations of increased psychosocial difficulties in perceiving and processing of social cues in A-allele carriers due to increased sensitivity toward negative parenting. In contrast, G-allele carriers display better coping strategies and therefore show more empathy in general and react more prosocially, independent of perceived rejection.

Together, this suggests that allelic variation at the rs53576 locus may confer differential susceptibility to the development of psychopathological signs and symptoms, depending on the quality of early family conditions (31) [for review see (32)]. The question as to whether genetic variation of the *OXTR* is associated with empathy in adults who experienced childhood adversity has been not been addressed so far.

The present study thus aimed to investigate the association between polymorphic variation of the SNP rs53576 and empathy for somatic and psychological pain and its interaction with childhood maltreatment. The sample included healthy participants as well as patients with BPD who were highly affected by the experience of negative events during childhood, including neglect and abuse. We hypothesized a significant interaction of genotype and empathy with A-allele carriers being more susceptible to childhood maltreatment than GG homozygotes. We further expected to find this effect being more pronounced in BPD compared to healthy controls.

## Materials and methods

### Participants

For the current study we recruited 148 female in-patients with BPD from the LWL-University Hospital Bochum and 154 female healthy control participants (total *n* = 302) via advertisement. We recruited exclusively female participants because the task utilizes pictures showing a woman in painful situations. The age of participants was between 18 and 50 years. BPD patients were unaffected by neurological illness and other severe somatic disorders. None of the participants was pregnant. Comorbid disorders and medication of the BPD patients are listed in Table 1. The study was approved by the local ethics committee of the medical faculty of the Ruhr-University Bochum (project number 4639-13). The authors assert that all procedures contributing to this work comply with the ethical standards of the relevant national and institutional committees on human experimentation and with the Helsinki Declaration of 1975, as revised in 2008. All participants gave their full informed consent in writing.

**Table 1:** Comorbid disorders and medication in the group of patients with BPD.

	***n***	**%**
**COMORBID DISORDERS OF PATIENTS WITH BPD**		
Depressive episode	79	53.4
Post-traumatic stress disorder	23	15.5
Phobic/ anxiety disorder	9	6.1
Obsessive compulsive disorder	1	0.7
Cannabis misuse	16	10.8
Alcohol misuse	21	14.2
Eating disorder	12	8.1
Other substance misuse	5	3.8
**MEDICATION**		
Without regular medication	61	42.2
Antidepressant	52	35.1
Antipsychotic	24	16.2
Antidepressant and antipsychotic drugs	24	16.2
Anticonvulsive substances	8	5.4
Other psychoactive drugs	8	5.4

### Questionnaires

General intelligence was estimated using the verbal intelligence test MWT-A (Mehrfachwahl-Wortschatz-Intelligenz-Test; 33). To assess the experience of maltreatment during childhood we used the short German version (34) of the Childhood Trauma Questionnaire (CTQ). The questionnaire contains 28 questions tapping into the history of emotional abuse, physical abuse, sexual abuse, emotional neglect, and physical neglect (35). The German version of the Interpersonal Reactivity Index (36), called “Saarbrücker Persönlichkeits-Fragebogen” (37) was used to measure empathic abilities in four scores, namely “perspective taking” (PT), “Fantasy” (FS), “empathic concern” (EC) and “personal distress” (PD).

### Social interaction empathy task

The Social Interaction Empathy Task (SIET) was developed by our group as described elsewhere (38, 39). In short, the paradigm investigates empathy for physical or somatic and psychological pain in one paradigm. Participants are asked to rate social interactions regarding pain intensity, with social interaction being either somatically painful, psychologically painful, or neutral, i.e., social situations that are free of pain or threat. Somatically painful pictures showed situations in which the woman was accidently hurt by another person, e.g., by cutting with a knife. In psychologically painful interactions the woman experienced social exclusion or rejection, as for example, by being abandoned by the partner. In one part they were asked to rate in a third-person perspective (e.g., how the woman in the picture feels) and in another part they should rate how they would feel themselves in these situations which we called first-person or self-perspective.

### Genotyping

The DNA samples were collected using Oragene OG-500 collection kits (DNA Genotek, Inc., Ottawa, ON, Canada) and by mouthwash with a commercially available mouthwash solution (Listerine). The DNA extraction was performed following the manufacturer's instructions of the Oragene Kit and an adapted version for the mouthwash samples, using a standard salting-out procedure Miller et al. (40). DNA concentration duplicate measures using BioTek microplate reader and Gen5™ software revealed a range of DNA concentrations between 10.0 ng/μl and 1089.7 ng/μl for the Oragene samples (mean concentration 278.3 ng/μl, SD 205.1 ng/μl), and 2.6 ng/μl and 876.8 ng/μl for the mouthwash samples (mean concentration 270.1 ng/μl, SD 209.7 ng/μl), indicating that both techniques led to sufficient DNA concentrations [comparison between collection methods *t*_(300)_ = 0.333, *p* = 0.739] that could be processed further by PCR. The DNA samples were subsequently diluted to a concentration of (20 ng/μL). The genotyping of *OXTR* rs53576 was conducted by real time PCR with fluorescence melting curve detection using the CFX384 Touch Real-Time PCR Detection System from Bio-Rad (Bio-Rad Laboratories, Inc., Hercules, CA 94547, USA). The primers were ordered from MWG Eurofins (Ebersberg, Germany) and the Taq qPCR Mastermix was obtained from Promega (Promega GmbH, Mannheim, Germany). The following primers flanking the rs53576 genomic region were used for the PCR reaction:

5′- CCCTGTTTCTGTGGGACTGA-3′ (forward), 5′-tggaaaggaaaggtgtacggg-3′ (reverse).

### Statistical analysis

Since the SIET is composed of two parts, i.e. two different perspectives (first and third-person perspective) and three pain condition (somatic pain, psychological pain, neutral), we chose a repeated measures ANOVA approach. Specifically, the ANOVA was conducted for the within-subject factors “condition” (somatic pain/ psychological pain/ neutral) and perspective (first-person perspective/ third-person perspective) and the between subject factors “group” (BPD/HC) and genotype (GG/ GA+AA). To control for “age” and “IQ” we included both variables as covariates in the ANOVA. An additional ANOVA was calculated for the three genotypes (GG/GA/AA) and can be found in the Supplemental Material. Results reported were Greenhouse-Geisser corrected values. For *post-hoc* comparisons, independent and dependent *t*-tests were performed and adjusted for multiple comparisons by Bonferroni correction.

For comparisons of questionnaires between groups independent two-sample *t*-tests were used. We further calculated partial correlations (controlling for age and IQ) for CTQ, IRI scores and the pain ratings of the SIET.

Moderation analyses were conducted by using the macro tool PROCESS developed by Hayes (41). These analyses were calculated for the independent variable CTQ total score (X), the dependent variable “pain rating” (psychological pain, somatic pain, neutral interactions; Y), the moderator (M), i.e. the rs53575 genotype and the covariates IQ and age. Correlations were calculated using partial correlations (with the factors age and IQ) for CTQ total score and subcores (*n* = 6) and the pain ratings for neutral and psychologically painful interactions, because the model was significant only for these two pain conditions. The results were Bonferroni-corrected due to multiple testing leading to a significance level of *p* < 0.0042 (*p* = 0.05/12).

To explore whether the data were in accordance with the differential susceptibility model, we further investigated the association of the independent variable (CTQ) and the moderator by correlation analyses and we calculated the differences of slopes of the associations between the two genotype groups.

## Results

### Self-rating questionnaires

There were significant differences in CTQ scores between groups, with more severe experiences of childhood maltreatment in patients with BPD. Patients also scored higher in the personal distress score of the IRI, whereas healthy controls scored higher in perspective taking and fantasy (**Table 3**). No difference was found for the empathic concern scale. For correlations among questionnaires, see Table 2.

**Table 2:** Correlations among IRI and CTQ questionnaires and behavioral results of the SIET (pain rating).

	**Interpersonal Reactivity Index**				**Childhood Trauma Questionnaire**					
	**Perspective taking**	**Fantasy**	**Empathic concern**	**Personal distress**	**Total score**	**Emotional abuse**	**Physical abuse**	**Sexual abuse**	**Emotional neglect**	**Physical neglect**
Pain rating physical pain	0.056	0.051	0.117	0.118	−0.021	0.005	0.006	0.010	−0.049	−0.108
Pain rating psychological pain	−0.050	0.121	0.0717	0.369^**^	0.215^**^	0.286^*^	0.083	0.119	0.180^*^	0.158^*^
Pain rating neutral	−0.120	−0.022	−0.032	0.151^*^	0.226^**^	0.263^**^	0.164^*^	0.120	0.173^*^	0.189^*^
IRI perspective taking					−0.229^**^	−0.273^**^	−0.121	−0.146^*^	−0.202^*^	−0.221^**^
IRI fantasy					−0.094	−0.034	−0.088	−0.066	−0.115	−0.058
IRI empathic concern					−0.098	−0.101	−0.091	−0.064	−0.154^*^	−0.083
IRI personal distress					0.450^**^	0.526^**^	0.264^**^	0.243^**^	0.390^**^	0.342^**^

Results reported are correlation coefficients (r) with significant correlations printed in bold and

* indicating results with p < 0.05 and

** *representing p ≤ 0.001. Correlations within questionnaires are not shown*.

### Genotypes

Genotype distributions were in Hardy-Weinberg equilibrium in the whole sample [χ2_(2)_ = 1.582; *p* = 0.453] and were distributed as follows: GG genotype *n* = 128 (42.4%), GA genotype *n* = 136 (45%); AA Genotype *n* = 38 (12.6%). In accordance to previous studies, we divided the sample into GG and A genotypes (GA+AA) [see (17, 27, 28, 42)] resulting in GG *n* = 128 (42.4%) and A carriers (GA+AA) *n* = 174 (57.6%). Additional analyses of behavioral results (rmANOVA and *post-hoc* tests) for GG, GA and AA groups separately are shown in the Supplemental Material section.

### Social interaction empathy task

Repeated measures ANOVA with the covariates IQ and age revealed main effects and interactions, similar to results reported in previous studies using the SIET in patients with BPD (38, 39). Specifically, we detected a three-way interaction of condition^*^group (BPD/HC) ^*^genotype [*F*_(1.85)_ = 3.46; *p* = 0.036]. *Posthoc* group comparisons (separately for BPD and HC) showed no difference between genotypes in regard of pain ratings of somatic pain, psychological pain and neutral interactions (Bonferroni corrected for multiple comparisons, i.e., (*p* = 0.05/3 = 0.017). *Posthoc* comparisons of genotype (GA + AA vs. GG separately) showed differences in pain intensity ratings between patients with BPD and HC only in A-allele carriers, whereas no differences occurred in GG homozygotes. Specifically, patients with BPD who carried at least one A-allele rated psychologically painful and neutral interactions as more intense than controls [psychological pain rating BPD *M* = 6.30 *SD* = 1.53; HC *M* = 4.97 *SD* = 1.60; *t*_(168)_ = 5.56, *p* < 0.001; neutral pain rating BPD *M* = 1.81 *SD* = 0.87; HC *M* = 1.32 *SD* = 0.40; *t*_(168)_ = 4.70, *p* < 0.001], a pattern that was also observed for the whole group (see Table 3).

**Table 3:** Results of Age, IQ scores, self-rating questionnaires (IRI, Interpersonal Reactivity Index; CTQ, Childhood Trauma Questionnaire) and the Social Interaction Empathy Task (SIET) in patients with BPD (BPD) and healthy controls (HC).

	**BPD**		**HC**				
	***M***	***SD***	***M***	***SD***	***t***	***p***	***df***
AGE	27.7	7.9	24.6	5.4	4.0	< 0.001	259.2
IQ	101.8	17.0	108.2	17.0	−3.14	0.002	277.0
**IRI**							
Perspective taking	14.2	5.8	19.2	4.3	−8.11	< 0.001	227.2
Fantasy	16.6	7.0	19.0	5.4	−3.21	< 0.002	232.3
Empathic concern	20.0	5.4	20.2	4.3	−0.33	0.744	236.4
Personal distress	21.3	4.5	12.8	5.0	14.95	< 0.001	273.3
**CTQ**							
Emotional abuse	17.1	5.8	7.6	3.7	14.92	< 0.001	189.1
Physical abuse	9.6	5.8	5.6	3.7	7.39	< 0.001	142.7
Sexual abuse	9.4	5.9	5.3	1.2	7.43	< 0.001	121.2
Emotional neglect	17.3	5.7	8.7	4.0	13.33	< 0.001	202.5
Physical neglect	10.3	4.2	6.3	2.3	8.91	< 0.001	168.9
Total score	63.7	19.5	33.9	10.8	14.36	< 0.001	173.6
**SOCIAL INTERACTION EMPATHY TASK**							
**Combined perspectives**							
PR psychological pain	6.2	1.6	5.2	1.7	5.62	< 0.001	291
PR somatic pain	6.7	1.6	6.6	1.5	0.51	0.609	291
PR neutral	1.8	0.9	1.4	0.6	4.74	< 0.001	241.1
**First-person perspective**							
PR psychological pain	6.6	1.6	5.2	1.8	6.77	< 0.001	291
PR somatic pain	6.3	1.8	6.4	1.8	−0.16	0.877	291
PR neutral	1.9	1.1	1.4	0.6	5.10	< 0.001	221.7
**Third-person perspective**							
PR psychological pain	5.8	1.7	5.1	1.6	3.70	< 0.001	292
PR somatic pain	7.1	1.5	6.8	1.5	1.28	0.201	292
PR neutral	1.8	0.8	1.4	0.6	3.70	< 0.001	261.7

*The table shows the average results (M), standard deviations (SD) and the statistics of t-test between groups. Results of the SIET are reported as pain rating results (PR) for both perspectives combined and for the first-person perspective and third-person perspective separated*.

In addition, we detected an condition^*^genotype interaction [*F*_(1.85)_ = 4.06; *p* = 0.020], however, *posthoc* comparisons failed to show significant differences for pain ratings between genotype groups.

Another interaction of condition with age appeared [*F*_(1.85)_ = 9.79, *p* < 0.001], indicating that age was positively correlated with pain rating of somatic pain (*r* = 0.196, *p* = 0.001), but not with the other pain conditions. For correlations of pain ratings with questionnaires see Table 2.

### Moderation analyses and correlations

Based on our previous findings indicating a crucial role of early aversive experiences in empathy for pain performance, especially psychologically pain (38, 39), we sought to examine whether this association was influenced by genetic variation of the *OXTR*. To study the effects of genotype on empathy in relation to early adversity, we performed a moderation analysis with the independent variable CTQ total score (X), the dependent variable pain rating during the empathy task (psychological pain, somatic pain, neutral interactions; Y) and the moderator (M) the rs53575 genotype. Since we did not detect any effect of “group” (i.e., diagnosis) in the ANOVA, we included all participants into all further analyses.

With regard to psychological pain, the overall model was significant [*F*_(5, 216)_ = 3.35, *p* = 0.006, *R*^2^ = 0.0720], as was the interaction of CTQ by genotype: Interaction *b* = −0.0214, *t*_(216)_ = −2.10, *p* = 0.037. These results suggest that genotype has a moderating effect on the influence of childhood maltreatment on empathic reactions to psychological pain. Correlation analyses support this assumption by showing correlations of CTQ scores and pain rating only in A-carriers.

In A-allele carriers (AA and GA genotypes pooled), CTQ subscales and the total score correlated positively with ratings of psychological pain (CTQ total score *r* = 0.348, *p* < 0.001; emotional abuse *r* = 0.422, *p* < 0.001; emotional neglect *r* = 0.315, *p* < 0.001 and physical neglect *r* = 0.274, *p* = 0.002). In contrast, no correlation emerged in GG homozygotes.

**Figure 1:**
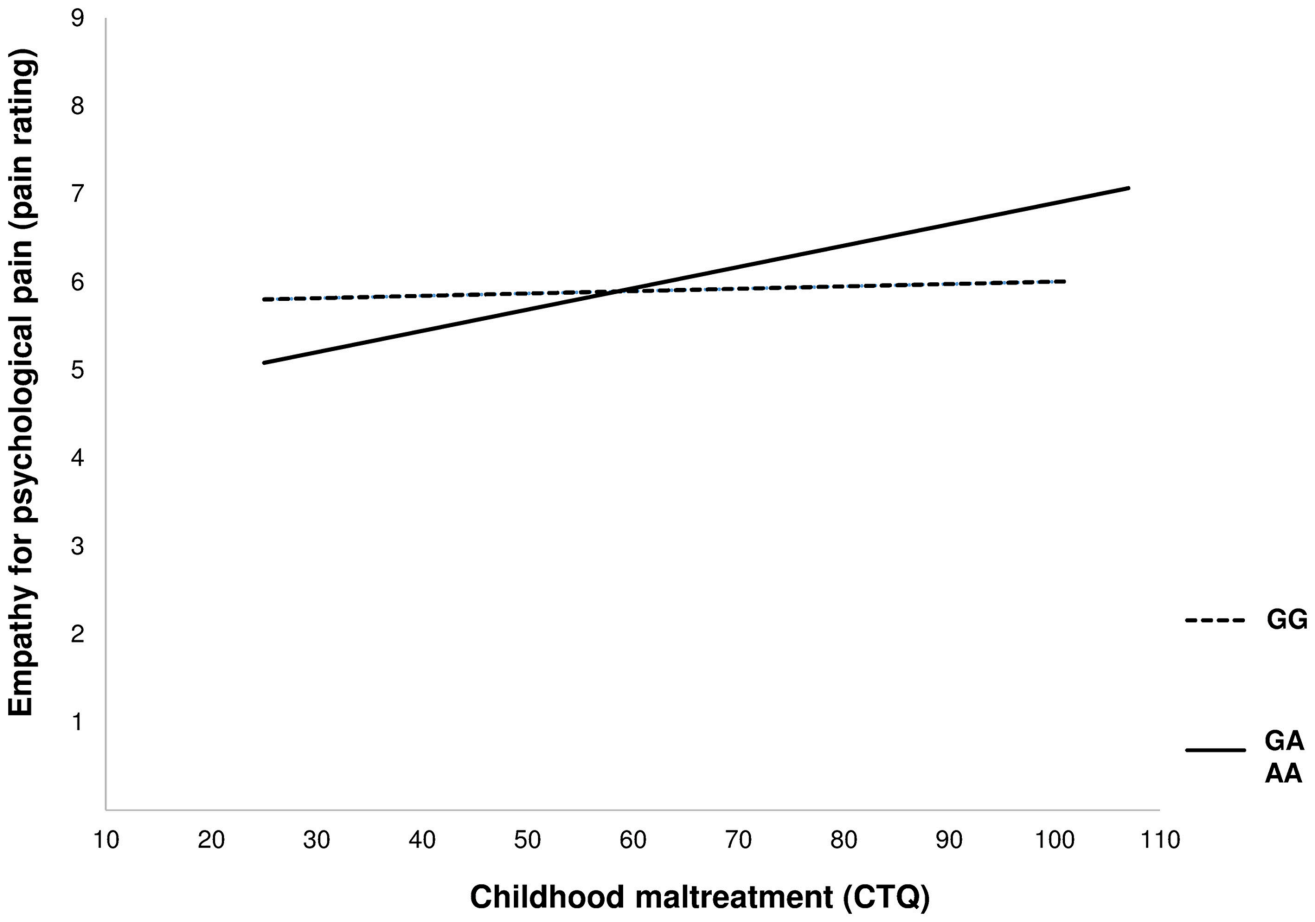
Depiction of the moderating effect of the rs53576 genotype on the association of childhood maltreatment with empathy for psychological pain.

In order to examine the assumption of differential susceptibility with regard to empathy for psychological pain, we tested if the predictor was associated with the moderator. In support of the differential susceptibility model, no direct correlation emerged between CTQ score and genotype [*r* = −0.075, *p* = 0.260; for more details see (43)]. Notably, only the simple slope of the association of CTQ with pain rating of psychological pain (Figure 1 solid line) in the GA+AA group differed significantly from zero (GA+AA *b* = 0.027, *SE* = 0.0069, *p* = 0.001, GG: *b* = 0.0056, SE = 0.0080 *p* = 0.4851). Moreover, the slopes between genetic groups differed significantly (GA+AA vs. GG: *t* = 2.88, *p* = 0.004).

As regards somatic pain, the overall model did not reach significance, with *F*_(5, 216)_ = 1.354, *p* = 0.243, *R*^2^ = 0.0304, and the interaction of genotype and CTQ did not reach significance [*b* = −0.0087, *t*_(216)_ = −0.878, *p* = 0.381]. The slopes also did not differ from 0 (GA+AA *b* = 0.0019, *SE* = 0.0067 *p* = 0.776; GG *b* = −0.0067, *SE* = 0.0078, *p* = 0.386) and from each other (GA+AA vs. GG: *t* = 1.19, *p* = 0.236).

For neutral social interaction, the model was significant with *F*_(5, 216)_ = 3.78, *p* = 0.003, *R*^2^ = 0.0804, whereas the interaction of genotype and CTQ was not significant (*b* = −0.0044, *t*_(216)_ = −0.991, *p* = 0.323). Here, ratings of neutral social interactions correlated with CTQ scores (CTQ total score *r* = 0.319, *p* < 0.001; emotional abuse *r* = 0.345, *p* < 0.001; physical abuse *r* = 0.281, *p* = 0.002; emotional neglect *r* = 0.260, *p* = 0.004 and physical neglect *r* = 0.279, *p* = 0.002), only in A-allele carriers. No correlation emerged in GG homozygotes. In addition, only the slope for GA+AA genotypes differed from 0 (GA+AA *b* = 0.010, *SE* = 0.0030 *p* = 0.006; GG *b* = 0.0059, *SE* = 0.0035, *p* = 0.089) but difference between the slopes failed to reach statistical significance (GA+AA vs. GG: *t* = 1.36, *p* = 0.176).

## Discussion

The present study aimed to examine the role of a common oxytocin receptor gene variant and its interaction with childhood trauma in empathy for pain. In our sample, comprising equal numbers of patients with BPD and non-affected controls, we found a moderating effect of genotype on the association of childhood maltreatment with empathy for psychological pain. In A-allele carriers of rs53576, childhood trauma was differentially associated with empathy for psychological pain, which was not apparent in GG carriers. Specifically, as tentatively suggested from the cross-over of the regression lines, the type of gene-environment interaction seems to be consistent with the idea of differential susceptibility.

The concept of differential susceptibility implies that individuals with vulnerable genotypes are more susceptible for negative as well as positive environmental influences (43). Put another way, individuals experiencing parental warmth and emotional availability would be at lower risk than average to develop psychopathological signs and symptoms. In contrast, the diathesis-stress model claims that individuals carrying a risk-genotype show impaired functioning when experiencing adversity, but not lower-than-average risk under favorable conditions [(44), for a comprehensive discussion, see (45)].

In our own study, childhood maltreatment in A-allele carriers was associated with higher empathy for psychological pain, whereas low scores on the CTQ were related to less empathy for psychological pain. In contrast, GG homozygotes seemed to be unresponsive to early maltreatment with regard to empathy for psychological pain. The differential effect of adverse experiences during childhood in A-allele carriers is particularly remarkable, because, as Belsky et al. (31) noted, the absence of maltreatment during childhood (indicated by low CTQ scores) does not reflect particularly high parental emotional responsivity.

This interpretation is consistent with our previous work demonstrating an association of childhood maltreatment with empathy for psychological pain in patients with BPD, which was mediated by alexithymia (38). Importantly, we found that patients with BPD showed a disproportionally higher empathy for psychological pain relative to controls. We argued that this finding could reflect an emotional over-involvement due to projection mechanisms in the clinical group, because patients with BPD are known to be particularly sensitive to feelings of rejection or social exclusion [e.g., (46)]. In accordance with this interpretation, Hammen et al. (27) reported elevated levels of BPD symptomatology in youths when grown up with a depressive mother and less symptoms when raised under positive conditions in A-allele carriers. GG homozygotes show average levels of BPD symptoms independent of family quality. In another study, an association of unsupportive parenting and coping strategies emerged, which was shown to affect the development of depressive symptoms in A-allele carriers (28). GG carriers are therefore suggested to be more unresponsive toward early adversity. Poulin demonstrated that greater perceived threat predicted less prosocial behavior in terms of charitable activity only in A-allele carriers, which may indicate that GG individuals buffered against this negative association (22, 47). These results point toward an effect of environmental influences on social functioning depending on *OXTR* variants with A-allele carriers being more responsive (48). It therefore seems plausible to suggest that A-allele carriers who are exposed to early adversity such as maltreatment may be more at risk to develop unfavorable coping strategies, which in turn affects responsivity to social stimuli such as empathy for psychological pain.

Our findings are at odds with other studies showing an association of experiences of childhood maltreatment with depression symptoms in carriers of at least one G-allele (49). They further reported higher scores of depression in carriers of the G-allele when compared to AA homozygotes which were mediated by distrust/cynicism, which in turn may results from aversive experiences. Bradley also reported disorganized attachment patterns and higher levels of emotional dysregulation, when exposed to childhood trauma, in GG homozygotes compared to A-allele carriers in a sample of African Americans (50).

It is unclear why such profound differences between studies exist. Plausible factors impacting on the results include differences in the nature of early adversity (that is, neglect and abuse need to be disentangled), differences in sex, age, and ethnicity, as well as methodological issues, which includes the formation of groups according to genotype (GA/AA vs. GA/GG). In the present sample comprising a clinical group of female patients with BPD and female healthy participants, the A-allele moderated the association of childhood maltreatment on empathy for psychological pain. Moreover, the interaction between group by genotype by pain condition showed that differences between patients with BPD and healthy controls were only significant in the A-allele group for ratings of psychological pain and neutral interactions. This result suggests that individuals carrying at least one A-allele are more susceptible to environmental variation, which may indirectly be involved in the development of emotional instability. Previous research has suggested that variations of the *OXTR* may be involved in BPD (32, 51), however, little is known about the particular contribution of the rs53576 polymorphism in relation to BPD. Thus, further research is necessary to clarify its role, especially since the oxytocin system itself is suggested to play a key role in the development of BPD. In addition to the SNP examined here, the idea that other genes conveying differential susceptibility is relevant in the etiology of BPD warrants further examination (52, 53).

A considerable number of studies have reported associations between rs53576 and socio-behavioral phenotypes, however, the mechanism through which this intronic SNP might exert an effect is unknown. Several options have been discussed. For example, intronic genetic variants may act as enhancer elements that affect gene expression (54) or binding of transcriptional factor to these regions affect gene regulation (55). Furthermore, effects might be due to linkage disequilibrium with functional SNPs in the regulatory region (56).

The present study has several limitations. First, only females were investigated. Previous work has demonstrated differences between men and women in regard of *OXTR* polymorphisms (26, 57), which highlights the importance of further studies of sex differences concerning differential susceptibility. Second, our groups differed in age and IQ which is another major limitation.

To conclude, the present study demonstrates an association of childhood maltreatment and empathy for psychological pain which was mediated by rs53576 genotype, suggesting that the A-allele may convey genetic plasticity to environmental factors (31).

## Author contributions

VF: Study design, acquisition, analysis, interpretation of data, drafting the article, final approval of the version to be submitted; DM: Data acquisition, analysis, interpretation of data, revision of manuscript for important intellectual content, final approval of the version to be submitted; RK: Study design, interpretation of data, revision of manuscript for important intellectual content, final approval of the version to be submitted; MB: Study design, interpretation of data, drafting the article, revision of manuscript for important intellectual content, final approval of the version to be submitted; All authors have approved the final article.

### Conflict of interest statement

The authors declare that the research was conducted in the absence of any commercial or financial relationships that could be construed as a potential conflict of interest.
