# Supplementary material for: The OXTR Single-Nucleotide Polymorphism rs53576 Moderates the Impact of Childhood Maltreatment on Empathy for Social Pain in Female Participants: Evidence for Differential Susceptibility
Source: Front Psychiatry. 2018 Aug 8;9:359. doi: 10.3389/fpsyt.2018.00359 (PMC6092568; doi:10.3389/fpsyt.2018.00359)
Supplement: Supplementary file 1 [file Table_1.pdf]

Supplementary Material

Repeated measures ANOVA of empathy for pain in relation to polymorphic variation of the OXTR

Results: Social Interaction Empathy Task and OXT rs53576 in GG, GA and AA carriers

An ANOVA with three genotype groups (i.e. GG, GA and AA), including IQ and age as covariates, revealed similar results in comparison to the pooled data for A-allele carriers (i.e. AA and GA). That is, there was a significant condition*genotype interaction ($F(3.70) = 2.74$; $p = 0.032$). Posthoc tests indicated that AA homozygotes rated somatic pain as more intense compared to GG homozygotes (AA pain rating $M = 7.10$ $SD = 1.27$; GG pain rating $M = 6.46$ $SD = 1.65$; $t(79.52) = -2.55$, $p = 0.013$), although this finding did not survive Bonferroni correction for multiple testing.
Furthermore, no differences were found for comparisons of pain ratings between GA and AA carriers and between GG and GA carriers (psychological pain rating GA $M = 5.59$ $SD = 1.63$; AA $M = 5.58$ $SD = 1.63$; neutral pain rating GA $M = 1.60$ $SD = 0.76$; AA $M = 1.46$; $SD = 0.57$). Similar to results of GA+AA vs. GG genotypes, we found an interaction of condition with age ($F(1.85) = 9.64$; $p < 0.001$).
